# Estimating and testing haplotype–trait associations in non-diploid populations

**DOI:** 10.1111/j.1467-9876.2009.00673.x

**Published:** 2009-12

**Authors:** X Li, B N Thomas, S M Rich, D Ecker, J K Tumwine, A S Foulkes

**Affiliations:** 1 University of MassachusettsAmherst, USA; 2 Rochester Institute of TechnologyUSA; 3 University of MassachusettsAmherst, USA; 4 Makerere UniversityKampala, Uganda; 5 University of MassachusettsAmherst, USA

**Keywords:** Expectation–maximization algorithm, Genotype, Haplotype, Human immunodeficiency virus, Linear model, Malaria, Phenotype, Quasi-species, Strain

## Abstract

Malaria is an infectious disease that is caused by a group of parasites of the genus *Plasmodium*. Characterizing the association between polymorphisms in the parasite genome and measured traits in an infected human host may provide insight into disease aetiology and ultimately inform new strategies for improved treatment and prevention. This, however, presents an analytic challenge since individuals are often multiply infected with a variable and unknown number of genetically diverse parasitic strains. In addition, data on the alignment of nucleotides on a single chromosome, which is commonly referred to as haplotypic phase, is not generally observed. An expectation–maximization algorithm for estimating and testing associations between haplotypes and quantitative traits has been described for diploid (human) populations. We extend this method to account for both the uncertainty in haplotypic phase and the variable and unknown number of infections in the malaria setting. Further extensions are described for the human immunodeficiency virus quasi-species setting. A simulation study is presented to characterize performance of the method. Application of this approach to data arising from a cross-sectional study of *n*=126 multiply infected children in Uganda reveals some interesting associations requiring further investigation.

## 1. Introduction

Our investigation is motivated by a study of the human pathogenic species *Plasmodium falciparum*, the group of parasites that cause malaria. Here, interest lies in characterizing associations between genetic polymorphisms in the haploid parasite and clinical measures of severity of disease, such as red blood cell (RBC) count or the amount of parasite in plasma. In this setting, multiple infections can arise as a result of two or more singly infected mosquitoes taking blood meals from the same individual, an infected mosquito taking blood meals over several days or a single multiply infected mosquito taking a blood meal from an individual. These three settings are indistinguishable from a data analytic perspective and all result in multiple strains within a single human host. In general, the observed genotype data consist of the set of nucleotides at each location of the genome across the entire population of organisms within a single host. Thus, as in the human genetics setting, the specific alignment of these nucleotides on a single chromosome, which is called the haplotype, is generally unobservable. This constitutes the first analytic challenge.

The second challenge, rendering the infectious disease setting unique from human investigations, is that the number of infections is unknown and this number can vary across individuals. Combined, these two challenges serve as the motivation for our present research. Consider, for example, an individual who is infected by multiple parasites. Further suppose that the observed genotype for this individual is *Aa* at one site and *Bb* at a second site on the genome, where *A*, *a*, *B* and *b* are used to represent the observed nucleotide. In this simple case, there are four possible haplotypes: *h*_1_=(*A*,*B*), *h*_2_=(*A*,*b*), *h*_3_=(*a*,*B*) and *h*_4_=(*a*,*b*). The precise combination of these haplotypes within this individual is not observable. In a human population, the number of homologous chromosomes is fixed at 2 and therefore the truth could be (*h*_1_,*h*_4_) or (*h*_2_,*h*_3_). However, in the malaria setting, since the number of strains within each person is also unobserved, the number of copies of each haplotype is unknown. In this case, the true haplotype combination could be (*h*_1_,*h*_4_), (*h*_2_,*h*_3_), (*h*_1_,*h*_4_,*h*_4_) or (*h*_1_,*h*_1_,*h*_4_,*h*_4_), etc. and depends on whether the individual has two, three or four infections. Note that two distinct strains may have the same haplotype for the gene under consideration and thus we include, for example, (*h*_1_,*h*_4_) and (*h*_1_,*h*_4_,*h*_4_) as two distinct possibilities.

Several methods for characterizing population level haplotype frequencies and haplotype–trait associations in human populations have been described (Excoffier and Slatkin, 1995; Stephens *et al.*, 2001; Zaykin *et al.*, 2002; Stephens and Donnelly, 2003; Schaid *et al.*, 2002; Lake *et al.*, 2003; Lin and Zeng, 2006; Foulkes *et al.*, 2008). In this paper, we propose an extension of the EM approach for haplotype–trait association studies (Lake *et al.*, 2003; Lin and Zeng, 2006) for infectious disease settings. Here interest lies similarly in characterizing the relationship between genetic information and a trait; however, in the infectious disease context, the genetic information is typically measured on the infectious agent (such as a parasite or virus) rather than the human. In both cases, we assume that the trait is a host (human) level measurement.

In previous work, we described an expectation–maximization (EM) type of algorithm for estimating haplotype frequencies in the malaria setting that uses only the observed genotype data (Li *et al.*, 2007). This prior work, while extending the methods of Excoffier and Slatkin (1995) and Hill and Babiker (1995), does not take into account phenotypic or clinical information about the host. In this paper, we propose an EM-type algorithm that additionally takes into account information on a measured trait. This provides a comprehensive framework for simultaneous estimation of population haplotype frequencies and haplotype–trait associations. Thus the method that is presented represents an extension of Li *et al.* (2007) to incorporate trait information as well as an extension of Lake *et al.* (2003) and Lin and Zeng (2006) to the non-diploid setting.

An underlying premise motivating our research is that haplotypes may explain variability in a measured trait that is not fully captured by consideration of genotype data alone. In human genetic settings, haplotype-based investigations are important if the polymorphisms under consideration are in linkage disequilibrium with the true disease-causing variant but are not themselves causal. In the malaria settings, the specific combinations of nucleotides on a single strain may be relevant to protein production and, ultimately, to parasite fitness. The method that is presented herein provides the framework for evaluating these potential associations.

In Section 2, we describe an extension of the EM framework for estimation and inference under several models for the distribution of the number of infections. In Section 3, this approach is applied in a simulation study as well as to data arising from a cohort of *n*=126 multiply infected children from Uganda. Section 4 describes extensions for the human immunodeficiency virus (HIV) quasi-species setting in which multiple strains can arise from repeat infections though, more generally, this is a result of external pressures, such as treatment exposures. Finally, in Section 5 we provide a discussion of our findings.

## 2. Methods

We begin in this section by outlining our notation and the structure of the data. We then describe three approaches to estimation of the effect of haplotypes on a quantitative trait that each involve different assumptions about the distribution of the number of infections:

- we assume that the number of infections within a host is fixed at a constant *C*>0;
- we assume that this number follows a conditional Poisson distribution where we condition on the presence of at least one infection;
- we make no assumption about the distribution of the number of infections and estimate separately the probabilities of having exactly *c* infections where *c*=1,2,…,*C* for *C* sufficiently large.

Finally, a formal testing procedure is described.

### 2.1. Notation

Let **G**=(*G*_1_,…,*G*_*n*_) where *G*_*i*_ is the unphased (observed) multisite genotype for individual *i*. Further suppose that 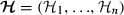 where 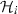 represents the combination of haplotypes within individual *i*. In general, 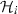 is not known and multiple values of 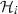 are consistent with *G*_*i*_. The set of all haplotype combinations that are consistent with *G*_*i*_ is denoted by 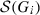. Let *h*_1_,…,*h*_*K*_ denote the *K* possible haplotypes over all observed individuals and define ***θ***=(*θ*_1_,…,*θ*_*K*_) where *θ*_*k*_ is the population frequency of *h*_*k*_. Now let **Y**=(*Y*_1_,…,*Y*_*n*_) where *Y*_*i*_ is the trait for *i*=1,…,*n*. We model **Y** by using the generalized linear model such that the expected value of *Y*_*i*_ is related to the linear predictor 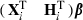 through a link function *g*: 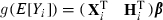
(1) where **X**_*i*_ is a vector of environmental or demographic covariates, including the intercept as the first element, **H**_*i*_ is a vector of numerical codes for 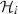 and ***β*** is the corresponding parameter vector. For a quantitative trait, *g*(·) reduces to the identity link. Since the haplotype combination for individual *i* is potentially unobserved, we consider all possible 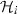 that are consistent with the observed genotype data, as described in Section 2.2. **H**_*i*_ can take many forms depending on the specific genetic model. For example, we may define **H**_*i*_ as a *K*×1 vector of indicators for the presence of a specific dominant haplotype in individual *i*. Alternatively, we can set the *k*th element of **H**_*i*_ equal to the number of copies of *h*_*k*_ in individual *i*, corresponding to an additive genetic model. Further discussion of formulations for this design matrix are given in Lin and Zeng (2006).

### 2.2. Estimation

In this section we describe the general EM framework for estimation, assuming a given distribution for the number of infections. We then elaborate on this algorithm for each of three distributional assumptions. First note that, for the generalized linear model framework, we assume that the probability density of **Y** is from an exponential family, given by 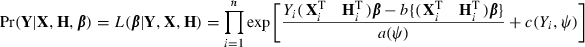
(2) where *a*, *b* and *c* are known functions, *ψ* is a scale parameter and in our setting **H** is unknown. The ambiguity in **H** renders the haplotype-trait association study a missing data problem and thus an EM-type algorithm is a natural choice for this setting. The EM algorithm, which was formalized by Dempster *et al.* (1977), involves first taking the conditional expectation of the complete-data log-likelihood (E-step), maximizing this with respect to the parameters of interest (M-step) and then iterating between these two steps until a convergence criterion has been met. In our setting, the observed data consist of **Y**, **X** and **G** and are denoted **X**^(obs)^, whereas the complete data consist of **Y**, **X**, **G** and 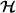 and are denoted **X**^(com)^. Let Φ be the parameters of interest, as described in each of the following sections. The complete-data likelihood for Φ is thus given by 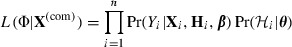
(3) where 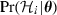 is the corresponding haplotype set probabilities for the *i*th individual. Notably, this likelihood assumes that the haplotype frequencies are independent of environmental or demographic information. In general, if departures from this assumption are tenable, a stratified analysis may be appropriate. As seen below, 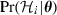 depends on the particular assumptions that are made with respect to the number of infections.

Let 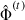 be the estimate of Φ derived from the *t*th iteration of the EM algorithm. Formally, we have that the expectation of the complete-data log-likelihood conditional on the observed data and the current parameter estimates is given by 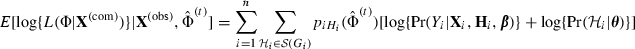
(4) where 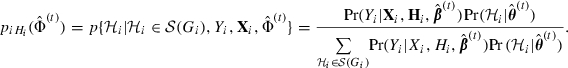
(5)

Next, we maximize the conditional expectation of the complete-data log-likelihood given in equation (4). It is straightforward to show that the (*t*+1)th estimate of Φ can be obtained by finding the root for the equations 
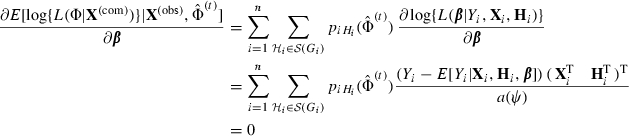
(6) and 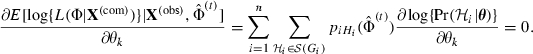
(7)

As noted in Lake *et al.* (2003) for the diploid setting, equation (6) reveals that the regression parameter ***β*** can be estimated via weighted regression, where the weights are the posterior probabilities of the haplotype sets for each individual, allowing us to use standard statistical software packages at this step. In the following subsections we describe estimation under specific assumptions for 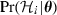. We assume convergence of the algorithm when 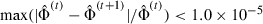. Alternatively, a convergence criterion can be based on the observed data likelihood, which is given by 
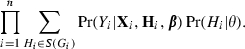


#### 2.2.1. Fixed number of infections

Let *δ*_*ik*_ denote the number of copies of haplotype *h*_*k*_ in the haplotype combination 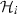. First suppose that there are exactly *C* strains in each individual where *C*>0, i.e. assume that each individual has exactly *C* infections, where *C* is some known positive integer. This implies that 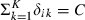, where *δ*_*ik*_ ranges from 1 to *C*. 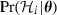 of equation (3) is thus given by 
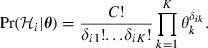
(8)

In this case, Φ=(***β***,***θ***). Plugging equation (8) into equation (7), we have 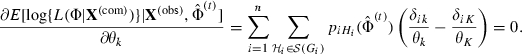
(9)

Resulting closed form solutions for 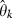 (see Appendix A.1) are given by 
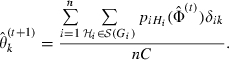
(10)

#### 2.2.2. Poisson assumption on the numbers of infections

In Section 2.2.1 we assumed that the number of infections is fixed; however, in general this number may be variable for each individual. In this section we relax this assumption and instead assume a Poisson distribution on the number of infections per individual, as described in Hill and Babiker (1995). Since data sets are generally comprised only of individuals with at least one detectable infection, the conditional Poisson model is considered. Let the Poisson model conditioning on at least one infection be given by 
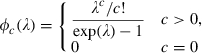
(11) where *φ*_*c*_(*λ*) is the probability of having *c* infections. In this case, Φ=(***β***,***θ***,*λ*). Since the number of strains *c*_*i*_ can be determined from 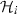, equation (8) for the haplotype combination probabilities is now replaced by 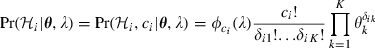
(12) where *c*_*i*_ is the number of infections for the *i*th individual. Estimation of ***θ*** proceeds similarly to the setting in which *C* is fixed. Straightforward calculation (see Appendix A.2) leads to closed form solutions for 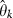 given by 
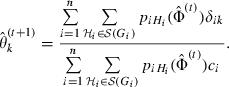
(13)

Estimation of *λ* is achieved by solving 
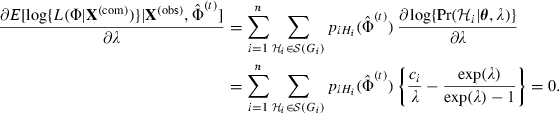
(14)

There is no closed form for 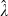 and a Newton–Raphson procedure can be employed. In this setting, the number of possible strains in an individual is not limited, which leads to an infinite sum in the E-step of the EM algorithm. In practice, we consider the number of strains to be limited by a large number (*C*) such that the probability of having more than *C* infections is small.

#### 2.2.3. Semiparametric approach

Finally, we consider the approach in which no assumptions are made about the distribution of the number of infections. In this approach, we estimate separately the probabilities of having exactly *c* infections where *c*=1,2,…,*C* for *C* sufficiently large. Let *q*_*c*_ be the probability of having *c* infections and define **q**=(*q*_1_,…,*q*_*C*_) and Φ=(***β***,***θ***,**q**). Equation (8) for the haplotype set probabilities is now replaced by 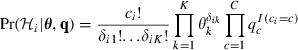
(15) where *I*(*c*_*i*_=*c*) equals 1 if *c*_*i*_=*c* and 0 otherwise. Estimation of **q** proceeds by solving 
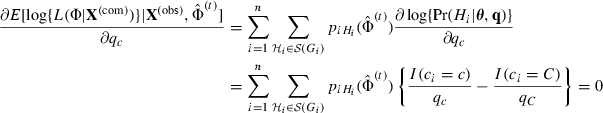
(16) and resulting closed form solutions (see Appendix A.3) for 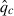 are given by
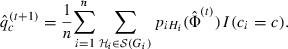
(17)

### 2.3. Inference

Wald tests are used to test hypotheses of haplotype–trait associations. To do this, estimates of the model parameters and the corresponding variance–covariance matrix are needed. Estimation of the variance–covariance matrix proceeds by inverting the observed information matrix, which is computed via Louis's method within the EM framework (Louis, 1982). An alternative approach is to approximate the observed information matrix with the empirical observed information matrix which can be computed by (Meilijson, 1989) 
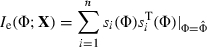
(18) where 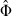 is the estimate of the parameters in the last EM iteration and *s*_*i*_(Φ) is the score function from the observed data likelihood for the *i*th individual. The score is given by (McLachlan and Krishnan, 1997) 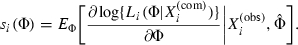
(19)

For example, under the fixed number of infections assumption, we have 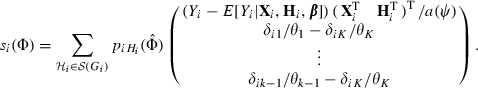
(20)

## 3. Data examples

In the following simulation study and real data example we focus on a quantitative trait for ease of presentation. In this case, *g*(·) of equation (1) is set equal to the identity link and we have the linear regression model 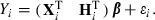
(21)

We further assume the *ɛ*_*i*_ are independent and normally distributed with mean 0 and variance given by *σ*^2^. Notably, this model assumes homoscedasticity and is therefore applicable when the standard deviation of the trait is constant over the values of **X** and **H**. In the real data example that is provided below, we have no biological reason to believe that there is a violation of this assumption though, in general, evaluation of the appropriateness of the homoscedasticity assumption can be achieved through close examination of residual plots.

### 3.1. Simulation study

To evaluate the performance of the methods that were described in Section 2, we conduct a simulation study and report the type 1 error rates (ERs) and power under each of the three models for the number of infections: fixed, Poisson and semiparametic. The simulation starts by generating the number of infections *c* for each individual. Under the fixed number model, the number of infections is set equal to a constant *C*. Under the Poisson assumption, *c* is generated randomly from a conditional Poisson distribution with assumed rate parameters *λ*=2 and *λ*=3. Finally, under the semiparametric approach, we assume that the number of infections *c* ranges from 1 to 4 with corresponding probabilities **q**=(0.3,0.3,0.2,0.2).

Next we simulate the haplotype combination for each individual on the basis of the multinomial distribution. Four haplotypes, which are given by *h*_1_=(*A*_1_,*B*_1_), *h*_2_=(*A*_1_,*B*_2_),*h*_3_=(*A*_2_,*B*_1_) and *h*_4_=(*A*_2_,*B*_2_), with corresponding population frequencies of ***θ***=(0.25,0.35,0.20,0.20), are assumed. The trait *Y* is generated by using random sampling with the error generated from a normal distribution. A single haplotype effect is assumed with an effect size ranging from 0.2 to 0.8. For simplicity of presentation, we let *σ*^2^=1 and vary *β*. In addition, we consider a model in which there is no haplotype effect, in which case the response is generated simply from a normal distribution with mean and variance equal to 1. In all cases, a dominant genetic model is assumed. For each configuration, *B*=200 data sets with sample sizes of *n*=500 are generated. Analysis is performed using genotype data and trait information only, i.e. we assume that the haplotypic phase and the number of infections are unknown and apply the methods that were described in Section 2.

Simulation results are provided in Table 1. Bias, coverage rates, power and ER are reported. Bias is defined as the absolute difference between the mean parameter estimates over the simulations and the true value. The estimated standard error of the parameter estimates based on the simulations is given by 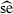. The parameter *β*_1_, the haplotype effect for the first haplotype *h*_1_=(*A*_1_,*B*_1_), is varied across the simulations. Power is defined as the proportion of simulations in which we detect the true haplotype effect. The ER is the proportion of simulations for which an incorrect haplotype is detected, averaged over the haplotypes that are assumed to have no effect.

**Table 1:** Simulation results for the dominant model under three assumptions†

	*β*_1_‡	( 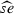 )§			*Coverage rates*§§			Power^*^	ER^**^
		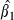	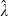	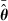	*β*_1_	*λ*	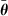		
*Fixed number model*									
*C*= 2	0.0	0.0038 (0.132)	—	0.0008 (0.016)	0.95	—	0.95	0.05	0.06
	0.2	0.0009 (0.138)	—	0.0005 (0.015)	0.96	—	0.95	0.35	0.07
	0.4	0.0060 (0.138)	—	0.0013 (0.015)	0.96	—	0.95	0.82	0.06
	0.6	0.0002 (0.126)	—	0.0003 (0.016)	0.95	—	0.95	0.99	0.06
	0.8	0.0016 (0.122)	—	0.0008 (0.015)	0.94	—	0.95	1.00	0.05
*C*= 3	0.0	0.0035 (0.180)	—	0.0007 (0.018)	0.94	—	0.94	0.08	0.07
	0.2	0.0122 (0.181)	—	0.0009 (0.017)	0.95	—	0.95	0.22	0.08
	0.4	0.0136 (0.187)	—	0.0006 (0.017)	0.95	—	0.95	0.59	0.08
	0.6	0.0265 (0.181)	—	0.0011 (0.017)	0.95	—	0.95	0.88	0.08
	0.8	0.0291 (0.177)	—	0.0004 (0.017)	0.95	—	0.94	0.97	0.07
*C*= 4	0.0	0.0128 (0.206)	—	0.0066 (0.019)	0.94	—	0.92	0.07	0.06
	0.2	0.0078 (0.223)	—	0.0037 (0.019)	0.97	—	0.94	0.20	0.09
	0.4	0.0443 (0.212)	—	0.0065 (0.020)	0.96	—	0.94	0.38	0.06
	0.6	0.0856 (0.185)	—	0.0048 (0.020)	0.93	—	0.95	0.62	0.07
	0.8	0.0627 (0.197)	—	0.0046 (0.018)	0.92	—	0.95	0.88	0.06
*Poisson model*									
*λ*=2	0.0	0.0098 (0.126)	0.0022 (0.111)	0.0025 (0.020)	0.96	0.94	0.94	0.04	0.05
	0.2	0.0011 (0.150)	0.0093 (0.105)	0.0011 (0.019)	0.95	0.95	0.95	0.41	0.05
	0.4	0.0001 (0.128)	0.0101 (0.089)	0.0013 (0.020)	0.96	0.96	0.97	0.87	0.06
	0.6	0.0240 (0.129)	0.0042 (0.116)	0.0018 (0.020)	0.94	0.98	0.96	1.00	0.03
	0.8	0.0160 (0.146)	0.0091 (0.104)	0.0012 (0.019)	0.96	0.95	0.94	0.99	0.05
*λ*=3	0.0	0.0022 (0.131)	0.0087 (0.123)	0.0017 (0.019)	0.96	0.97	0.94	0.04	0.03
	0.2	0.0312 (0.129)	0.0372 (0.124)	0.0027 (0.019)	0.95	0.96	0.95	0.44	0.04
	0.4	0.0002 (0.122)	0.0043 (0.137)	0.0017 (0.020)	0.94	0.96	0.95	0.91	0.05
	0.4	0.0055 (0.129)	0.0216 (0.137)	0.0009 (0.018)	0.93	0.96	0.94	0.99	0.06
	0.8	0.0120 (0.116)	0.0067 (0.126)	0.0024 (0.020)	0.97	0.96	0.94	1.00	0.06
			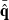			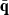			
*Semi-parametric model*									
	0.0	0.0034 (0.117)	0.0112 (0.033)	0.0024 (0.019)	0.95	0.79	0.96	0.05	0.03
	0.2	0.0082 (0.108)	0.0119 (0.030)	0.0027 (0.018)	0.94	0.85	0.95	0.38	0.06
	0.4	0.0024 (0.118)	0.0119 (0.029)	0.0018 (0.018)	0.96	0.81	0.96	0.94	0.06
	0.6	0.0321 (0.141)	0.0132 (0.032)	0.0027 (0.019)	0.97	0.83	0.96	1.00	0.04
	0.8	0.0015 (0.116)	0.0119 (0.032)	0.0007 (0.018)	0.96	0.83	0.95	1.00	0.05

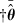
 and 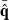 denote averaging across all 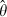
s and 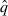
s respectively. 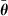 and 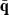 denote averaging across all *θ*s and *q*s respectively.

‡*β*_1_ is the effect of haplotype *h*_1_=(*A*_1_,*B*_1_) on *Y*.

§Bias is defined as the absolute difference between the mean of the estimate over the simulations and the true parameter value.

§§Coverage rate is defined as the proportion of simulations for which the true parameter value is within the corresponding 95% confidence interval.

*Power is the specific power for the haplotype effect of the first haplotype *h*_1_.

**ER is the type 1 error rate.

Under each of the three model assumptions and a range of haplotype effect sizes, the bias ranges from less than 0.001 to 0.086 and the coverage rates are between 0.92 and 0.97. This suggests that our algorithm results in reasonably well-calibrated interval estimates. As expected, the power for detecting the haplotype effect increases as the effect size increases from 0.0 to 0.8. In general, for samples of size of *n*=500, we achieve greater than 80% power to detect moderate effect sizes of greater than 0.40. Notably, however, we see a reduction in power and an increase in the bias for *β*_1_ as the number of infections (parasite strains) is increased from 2 to 4 under the fixed number assumption. This is likely to be the result of increased ambiguity associated with more possible haplotype combinations within an individual as the number of infections (*C*) increases.

To evaluate the performance of the proposed method when the number of infections violates model assumptions, we conduct several sensitivity analyses. First, we perform estimation by using the fixed approach, assuming that the number of infections is equal to 2, when in fact the probabilities of having *c* infections for *c*=1,…,5 are all equal to 0.2. The results are presented in Table 2, part (a). Comparing this with correct application of the semiparametric method (Table 1), we see a dramatic loss of power and a less severe, but noteworthy, decrease in coverage rates for both *β* and *θ*. In addition, the type 1 ER is substantially larger for *β*_1_≥0.4. Secondly, we perform estimation by using the fixed number approach, again assuming that the number of infections is equal to 2, when in fact the number of infections arises from a conditional Poisson distribution with *λ*=2. The results are presented in Table 2, part (b). Comparing these results with correct application of the Poisson approach with *λ*=2 (Table 1), we see a more dramatic decrease in coverage rates for both *β* and *θ*. In addition, a significant decrease in power and increase in the type 1 ER are observed for *β*≥0.2. These findings support the use of the more sophisticated modelling approaches in these settings.

**Table 2:** Sensitivity analysis to model misspecification

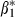	*Bias*			*Coverage rates*			*Power*	*ER*
	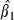	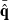	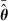	*β*_1_	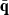	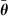		
*(a) Incorrect application of the fixed approach under semiparametric data*†								
0.0	0.0016 (0.133)		0.0332 (0.044)	0.95		0.90	0.03	0.04
0.2	0.0441 (0.165)		0.0334 (0.045)	0.93		0.92	0.22	0.04
0.4	0.0810 (0.187)		0.0366 (0.042)	0.92		0.86	0.59	0.12
0.6	0.0761 (0.251)		0.0303 (0.041)	0.92		0.88	0.88	0.22
0.8	0.1081 (0.329)		0.0214 (0.044)	0.93		0.93	0.95	0.30
*(b) Incorrect application of the fixed approach under Poisson-distributed data*‡								
0.0	0.0158 (0.178)		0.0640 (0.104)	0.93		0.99	0.08	0.07
0.2	0.1112 (0.175)		0.0850 (0.083)	0.89		0.92	0.13	0.09
0.4	0.1499 (0.187)		0.0985 (0.065)	0.91		0.64	0.30	0.16
0.6	0.2177 (0.219)		0.0972 (0.068)	0.86		0.68	0.65	0.25
0.8	0.3546 (0.353)		0.0722 (0.092)	0.87		0.98	0.83	0.40
*(c) Incorrect application of the conditional Poisson model*§								
0.0	0.0086 (0.115)	0.0492 (0.009)	0.0023 (0.022)	0.97	0.15	0.95	0.02	0.04
0.2	0.0110 (0.142)	0.0491 (0.009)	0.0019 (0.022)	0.95	0.14	0.95	0.37	0.07
0.4	0.0026 (0.129)	0.0489 (0.008)	0.0011 (0.020)	0.96	0.12	0.94	0.90	0.05
0.6	0.0039 (0.141)	0.0492 (0.008)	0.0010 (0.021)	0.94	0.13	0.96	0.99	0.05
0.8	0.0134 (0.102)	0.0492 (0.009)	0.0010 (0.020)	0.95	0.15	0.94	1.00	0.06
*(d) Incorrect application of the semiparametric approach under Poisson-distributed data*§§								
0.0	0.0113 (0.114)		0.0027 (0.019)	0.96		0.95	0.04	0.05
0.2	0.0166 (0.123)		0.0025 (0.021)	0.95		0.95	0.34	0.04
0.4	0.0316 (0.147)		0.0025 (0.020)	0.97		0.96	0.81	0.04
0.6	0.0191 (0.115)		0.0022 (0.021)	0.95		0.95	1.00	0.05
0.8	0.0233 (0.121)		0.0010 (0.019)	0.94		0.94	1.00	0.04

†The data are simulated assuming between one and five infections with equal probabilities of 0.20 whereas the estimation approach assumes *c*=2 fixed infections. See the caption for Fig. 1 for definitions of terms.

‡The data are simulated assuming a conditional Poisson distribution with *λ*=2, whereas the estimation procedure assumes *c*=2 fixed infections.

§The data are simulated assuming between one and five infections with equal probabilities of 0.20.

§§The data are simulated assuming a conditional Poisson distribution with *λ*=2. The number of infections is assumed to range from 1 to 10.

Next, we perform estimation by using the Poisson approach when in fact the probabilities of having *c* infections for *c*=1,…,5 are all equal to 0.2 and we present the results in Table 2, part (c). Here the modelling approach provides estimates of *λ* and, from this, we calculate 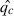 as 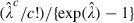. As expected under this type of model misspecification, the coverage rates for *q*_*c*_ are very low (0.12–0.15). Interestingly, the coverage rates for both *β* and *θ* remain at approximately 95% and the power and ER are reasonable, though slightly worse than using the correct model (Table 1). Finally, we evaluate performance in applying the semiparametric approach when the number of infections actually arises from a Poisson distribution with *λ*=2. These results are given in Table 2, part (d), and, as expected, we see a slight loss of power for the smaller effect sizes. For example, for an effect size of 0.4, the power of correctly using the Poisson approach is 0.87 (Table 1). Power for the semiparametric approach is estimated to be 0.81. Since we are not incorporating knowledge about the distribution of the number of infections the loss of power is expected.

### 3.2. Multiply infected children with malaria

Malaria is an infectious disease affecting millions of individuals globally. In fact, each year an estimated (1–3)-million people die as a result of infection with the human pathogenic *Plasmodium* species, the group of parasites that causes malaria (Breman, 2001). The majority of these deaths are in children under the age of 5 years and in resource-constrained settings since current treatment options are costly or unavailable (Greenwood *et al.*, 2005; Guerra *et al.*, 2008). Recent advances in sequencing technologies provide new opportunities for population-based genetic association studies to uncover complex relationships between genetic polymorphisms and measures of progression of disease. Ultimately, these discoveries may help to inform novel strategies for vaccine development.

One of the biggest challenges in characterizing genotype–trait associations in this setting arises from the fact that individuals can be infected simultaneously with multiple parasitic strains. In the present investigation, we apply an EM approach (see Section 2) to data arising from a cross-sectional study of *n*=126 malaria-infected children from Uganda. We focus on haplotypes in one polymorphic circumsporozoite protein (CSP) region (CSP-TH3) of the gene that encodes for a cellular adhesion domain of the CSP. The CSP facilitates adhesion of the parasite to liver cells, which is a critical initial step in its replication process in a human host (Zavala *et al.*, 1983; Hollingdale *et al.*, 1984). The goal of our analysis is to uncover haplotype associations with RBC count (log-transformed). The RBC count is a well-known diagnostic tool for detecting anaemia, which is a common and often lethal manifestation of malaria.

Data on 12 sites, 10 of which are polymorphic in our sample, are considered. Notably, sites that are constant across our data do not inform the analysis but are included for completeness. Across all individuals, we see up to three different nucleotides at a site and, within a single individual, one or two nucleotides are present at any given site. A total of 35 unique genotypes are observed in our data and a sample of the data is provided in Li *et al.* (2007). For computational purposes, the set of possible haplotypes is limited to those with estimated frequencies of greater than 0.01 where frequency estimates are obtained by using the approach of Li *et al.* (2007). We assume a Poisson distribution and apply the approach of Section 2.2.2. A dominant genetic model is assumed, as in the simulation study.

Estimated haplotype effects on the RBC and corresponding *p*-values for tests of the null hypotheses that these effects equal 0 are provided in Table 3. The *p*-values are unadjusted for multiple comparisons. Using a Bonferroni adjustment, *p*-values that are less than 0.05/14 = 0.0036 are considered significant at the 0.05-level. A significant association is observed between the RBC count and the three haplotypes numbered 8, 11 and 12. Interestingly, the effect of carrying at least one copy of haplotype 11 appears to increase the RBC count exp (0.344)=1.41-fold, suggesting a potential protective effect. In contrast, haplotypes 8 and 12 result in a lower RBC count with estimated decreases of exp (−0.484)=0.616-fold and exp (−0.137)=0.872-fold respectively. Notably, the estimated number of individuals with each of these haplotypes (which is given by 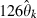) is small and further confirmatory research is required to make firm conclusions.

**Table 3:** Estimated haplotype effects for Uganda†

	*Unique haplotype*	*Estimated frequency* 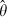	( 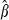 )	*Standard error*	*p-value*
1	T G A A C G C C G A G C	0.328	−0.108	0.099	0.278
2	T G A A C G C C G A G A	0.241	−0.066	0.092	0.471
3	T G A A C G C G A A G A	0.103	−0.032	0.106	0.762
4	T G A A C G C G G A G A	0.057	−0.148	0.150	0.324
5	T G G G T A C G G A G A	0.044	−0.257	0.151	0.089
6	T G G G C G C G G A G C	0.046	−0.081	0.240	0.737
7	T G A A C G C C A A G A	0.046	−0.023	0.165	0.891
8	T G G A C G C C G A G C	0.041	−0.484	0.133	<0.001‡
9	T G A A C G C G G A G C	0.034	0.200	0.583	0.731
10	T G G G C A C G G A G A	0.022	0.159	0.331	0.631
11	T G G G T G C G G A G A	0.011	0.344	0.008	<0.001‡
12	T G G A C G C C G A A T	0.005	−0.137	0.000	<0.001‡
13	T G G G C G A G A A G A	0.011	0.292	0.806	0.717
14	T G G A C G C C G A G A	0.009	0.206	2.031	0.919

†The results are based on a sample of size *n*=126 and assume a Poisson model for the number of strains per individual.

‡The haplotype effect on the RBC count is significantly different from 0 after applying a Bonferroni adjustment for multiple comparisons.

## 4. Further extensions for the quasi-species setting

In the methods that were described above for estimation of haplotype effects on a trait, we incorporate population level haplotype frequencies. These frequencies can be thought of as the amount of each parasite strain circulating in the mosquito population that infects humans. Importantly, we assume that the frequencies within individuals reflect these population level parameters. In other words, the probability of being infected with a given strain does not depend on prior infections and is equal to the proportion of this strain in the general population. Patients who are infected with HIV similarly host a population of viruses; however, the presence of such a quasi-species generally results from external pressures, such as drug exposures, rather than multiple repeat infections. As a result, the frequencies of each haplotype within an individual may not reflect the true population level frequencies. This is evidenced, for example, by the existence of latent reservoirs of resistant variants that rapidly emerge in the presence of a drug.

For this reason, rather than using population level haplotype frequencies in the HIV setting, we consider the probabilities that an individual in the target population carries a given haplotype. Although this distinction is subtle, it does require modification of the estimation approach that was described in Section 2. Again let *G*_*i*_ be the unphased (observed) multisite genotype for the *i*th individual where *i*=1,…,*n*. Further suppose that 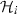 represents the combination of *unique* haplotypes within individual *i* where 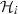 is generally unobservable and multiple values of 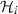 are consistent with *G*_*i*_. We emphasize unique here since, in the previously described approach, such a minimal set was not required, i.e. we are now interested in whether an individual carries a specific haplotype and not in the number of copies. Again, the set of all combinations that are consistent with *G*_*i*_ is denoted 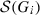 and *h*_1_,…,*h*_*K*_ denotes the *K* possible haplotypes over all observed individuals. Let ***α***=(*α*_1_,…,*α*_*K*_) where *α*_*k*_ is the probability that an individual carries at least one copy of *h*_*k*_ and define 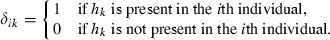
(22)

Under the model that is given in equation (1), the complete likelihood function can again be written as in equation (3) where 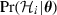 is replaced with 
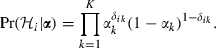
(23)

In this case, estimation of the regression parameter *β* proceeds as described above and an estimate of *α* is obtained by finding the root of the equation 
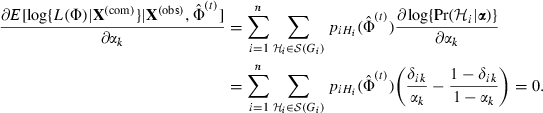
(24)

Resulting closed form solutions (see Appendix A.4) for 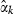 are given by 
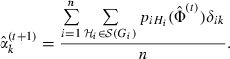
(25)

## 5. Discussion

In this paper, we describe an approach to estimate and test haplotype–trait associations between individuals with multiple strains of an infectious agent. Three approaches to modelling the number of infections were described in Section 2. The first, which involves fixing the number of infections to be a constant *C*, is presented since it represents a natural extension of the diploid setting, within which *C*=2 and our approach reduces to the EM method of Lake *et al.* (2003). Since in the infectious disease setting the number of infections is rarely known with certainty, this first approach may be more relevant to investigations of polyploidy organisms in which the number of homologous chromosomes is greater than 2, such as flatworms, goldfish, salmon and a variety of ferns and flowering plants. Note that the assumption of independent segregation that is made in equation (8) needs to be addressed specifically for each of these settings.

Our simulation study suggests that application of the Poisson approach, when in fact the numbers of infections are *c*=1,…,5 with equal probabilities, results in reasonable power and type 1 ERs but substantial bias in these probability estimates. The semiparametric approach performs reasonably well under the Poisson model with a slight loss of power. Incorrect application of the fixed number approach leads to more substantial losses of power, reductions in coverage rates and increases in type 1 ERs. Applications of the correct models lead to reasonable power and control of type 1 ERs.

Coupled with this investigation is the need for appropriate methods for controlling type 1 ERs in the context of multiple comparisons. In Section 3.2, we applied a Bonferroni correction to assess significance. Alternative single-step and step-down methods that are based on the false discovery rate and that account for the correlated nature of these tests (Benjamini and Hochberg, 1995; Benjamini and Yekutieli, 2001; Storey and Tibshirani, 2003) are also tenable. In addition, further consideration of resampling-based approaches and related extensions (Westfall and Young, 1993; Pollard and van der Laan, 2004; Foulkes and DeGruttola, 2007) may be appropriate. Extensions of the mixed effects modelling approaches that were developed originally for the diploid setting (Foulkes *et al.*, 2007, 2008) would offer a single degree of freedom omnibus test for association across all haplotypes.

Notably, our analysis is limited to data arising from individuals who visited one of the designated clinics. This may lead to ascertainment bias for several reasons, including that the individuals under study exhibited symptoms that were sufficiently severe to warrant at least one visit to the doctor. This is a potential limitation of the method that is described herein. Specifically, a population level prevalence greater than 0 of infection by a strain that results in mild symptoms may result in overestimation of the frequencies of haplotypes that lead to more severe symptoms.

Application of this EM approach to a small cohort of children in Uganda revealed three potentially informative haplotypes within the CSP region of the parasite genome. In general, characterizing the association between polymorphisms in the parasite genome and measured traits in an infected human host may provide greater insight into disease aetiology and help to inform new strategies for treatment and vaccine development efforts. Drawing meaningful biological and clinical conclusions, however, will require further analysis. Specifically consideration of host level factors, such as host genetic profile and clinical or demographic features, may be warrented. The methods that are described herein provide a general framework and the analytic tools to investigate such associations under several models of association and models for the numbers of infections.

## Appendix A

### A.1. Estimation under fixed number of infections

Note that the sum of the population level haplotype frequencies must equal 1, so we have . Equation (9) is then given by

for *k*=1,…,*K*−1 or, equivalently,

(26)

Note that all the elements of the vector on the right-hand side of equation (26) are equal. Therefore, we can set the first element of the vector on the left-hand side of equation (26) equal to each of the remaining elements of this vector, which yields

(27)

Thus we can derive an estimate of *θ*_1_ and use equation (27) to find estimates of *θ*_*k*_ for *k*=2,…,*K*−1. From the first element of equation (26) we have

or equivalently

(28)

Finally, since  and , equation (28) yields

### A.2. Estimation under Poisson assumption

Under the Poisson assumption, we have  and therefore equation (28) is written

resulting in

### A.3. Estimation for semiparametric approach

Note that the sum of the *q*_*c*_ must equal 1, so we have  and equation (16) is given by

for *c*=1,…,*C*−1. Using the same approach as described in Appendix A.1, we have

(29)

### A.4. Estimation for quasi-species setting

From equation (24), we have

or equivalently

(30)

Since , equation (30) yields
